# Clinical and In Vitro Studies on Impact of High-Dose Etoposide Pharmacokinetics Prior Allogeneic Hematopoietic Stem Cell Transplantation for Childhood Acute Lymphoblastic Leukemia on the Risk of Post-Transplant Leukemia Relapse

**DOI:** 10.1007/s00005-015-0343-0

**Published:** 2015-06-04

**Authors:** Joanna Sobiak, Urszula Kazimierczak, Dariusz W. Kowalczyk, Maria Chrzanowska, Jan Styczyński, Mariusz Wysocki, Dawid Szpecht, Jacek Wachowiak

**Affiliations:** Department of Physical Pharmacy and Pharmacokinetics, Poznan University of Medical Sciences, Święcickiego 6, 60-781 Poznań, Poland; Department of Cancer Immunology, Poznan University of Medical Sciences, Poznań, Poland; Department of Pathophysiology, Faculty of Medical Sciences, University of Warmia and Mazury, Olsztyn, Poland; Department of Pediatric Hematology and Oncology, Collegium Medicum, Nicolaus Copernicus University, Bydgoszcz, Poland; Department of Pediatric Oncology, Hematology and Transplantology, Poznan University of Medical Sciences, Poznań, Poland

**Keywords:** Etoposide, Conditioning, Hematopoietic stem cell transplantation, Graft versus leukemia, Pediatric acute lymphoblastic leukemia

## Abstract

The impact of etoposide (VP-16) plasma concentrations on the day of allogeneic hematopoietic stem cell transplantation (allo-HSCT) on leukemia-free survival in children with acute lymphoblastic leukemia (ALL) was studied. In addition, the in vitro effects of VP-16 on the lymphocytes proliferation, cytotoxic activity and on Th1/Th2 cytokine responses were assessed. In 31 children undergoing allo-HSCT, VP-16 plasma concentrations were determined up to 120 h after the infusion using the HPLC–UV method. For mentioned in vitro studies, VP-16 plasma concentrations observed on allo-HSCT day were used. In 84 % of children, VP-16 plasma concentrations (0.1–1.5 μg/mL) were quantifiable 72 h after the end of the drug infusion, i.e. when allo-HSCT should be performed. In 20 (65 %) children allo-HSCT was performed 4 days after the end of the drug infusion, and VP-16 was still detectable (0.1–0.9 μg/mL) in plasma of 12 (39 %) of them. Post-transplant ALL relapse occurred in four children, in all of them VP-16 was detectable in plasma (0.1–0.8 μg/mL) on allo-HSCT day, while there was no relapse in children with undetectable VP-16. In in vitro studies, VP-16 demonstrated impact on the proliferation activity of stimulated lymphocytes depending on its concentration and exposition time. The presence of VP-16 in plasma on allo-HSCT day may demonstrate an adverse effect on graft-versus-leukemia (GvL) reaction and increase the risk of post-transplant ALL relapse. Therefore, if 72 h after VP-16 administration its plasma concentration is still above 0.1 μg/mL then the postponement of transplantation for next 24 h should be considered to protect GvL effector cells from transplant material.

## Introduction

Acute lymphoblastic leukemia (ALL) is the most often pediatric malignancy (28 %) as well as the most often indication (approximately 30 %) to allogeneic hematopoietic stem cell transplantation (allo-HSCT) in children (Inaba et al. 2013; Peters 2012). Allo-HSCT significantly improves the results of conventional chemotherapy in children with very high-risk or relapsed ALL, but does not guarantee the sustained remission. The failure of allo-HSCT may occur due to the various complications related to the transplantation procedure, but mainly due to the post-transplant ALL relapse, especially in children transplanted in the second or subsequent complete remission (CR) (Locatelli et al. 2013; Mehta and Davies 2008; Raetz and Bhatla 2012). Therefore, for children with ALL further optimization of the conditioning regimen antileukemic effect is still required, e.g. by pharmacokinetic studies of the drugs used in conditioning (Hartmann and Lipp 2006; Liliemark et al. 1996; Mross et al. 1996; Würthwein et al. 2002). The aim is to establish a myeloablative regimen characterized by great drug exposure and minimal risk of life-threatening organ toxicity (Mross et al. 1996).

One of the most frequently used conditioning regimens prior to allo-HSCT in children suffering from ALL consists of fractionated total body irradiation (FTBI) at a total dose of 12 Gy, with the dose reduction in the lungs to 9 Gy, and high-dose etoposide (VP-16) at a single dose of 60 mg/kg administered as a 4 h infusion 72 h prior to allo-HSCT (Blume et al. 1987; Dopfer et al. 1991; Peters et al. 2015). This preparative regimen was for the first time implemented by Blume et al. (1987), and since 1991, when the results achieved in children with ALL in second CR (CR2) were published by Dopfer et al. (1991), has been applied to children above 2 years of age in BFM centers (International Berlin–Frankfurt–Monachium Study Group).

The VP-16 plasma concentration, 72 h after infusion of the agent, e.g. on day of allo-HSCT, is still to be defined. Some attempts, however, were made in vitro (Krüger et al. 1995; Littlewood et al. 1985; Rodman et al. 1994; Schwinghammer et al. 1993) and in vivo studies (Rodman et al. 1994). Rodman et al. (1994) observed a prolonged engraftment only in children in whom plasma VP-16 concentration on the autologous HSCT (auto-HSCT) day was greater than 5.00 μg/mL and VP-16 systemic clearance (CL_s_) was lower than 15 mL/min/m^2^. Following the results of the in vitro study performed by Schwinghammer et al. (1993), auto-HSCT should be performed when VP-16 concentration is lower than 0.30 μg/mL. Other in vitro study (Mross et al. 1994) showed that VP-16 concentration of 0.01 μg/mL caused 50 % inhibition of the progenitor cells forming granulocyte–macrophage colony (CFU-GM) in the protein-free medium. As VP-16 is strongly protein-bound, in the presence of the proteins the concentration of VP-16 causing 50 % inhibition of CFU-GM growth increased to 0.38 μg/mL. Although CFU-GM represents only a part of hematopoietic stem cells, it was shown that 50 % inhibition of CFU-GM correlates with longer bone marrow aplasia. Referring to the mentioned studies, further studies are required to establish the safe level of VP-16 concentration which may be present in plasma on the allo-HSCT day without causing any toxic damage to the transplanted allogeneic hematopoietic cells and to the immune cells present in the transplanted material, responsible for graft-versus-leukemia (GvL) effect (Kolb 2008).

Therefore, the aim of our study was to test the influence of VP-16 concentrations, present on the allo-HSCT day, on the incidence of post-transplant ALL relapse and on the probability of leukemia-free survival (pLFS) in children conditioned to allo-HSCT. We also assessed the in vitro influence of VP-16 on the proliferative and cytotoxic activity of T lymphocytes, considered as the potential effector cells of GvL reaction, stimulated with anti-CD3 and anti-CD28 antibodies, and on Th1/Th2 cytokine responses (Kolb 2008; Porter et al. 2006). We expect that the results of this study may help to optimize the conditioning regimen prior to allo-HSCT in children with ALL and improve the long-term treatment results.

## Materials and Methods

### Clinical Study

#### Patients and Allo-HSCT Characteristics

Thirty-one children (12 females, 19 males) aged 6–18 years, including 29 with ALL [16 in first CR (CR1), 12 in CR2, 1 in third CR (CR3)] and two with non-Hodgkin’s lymphoma (NHL) grade IV (1 in CR1, 1 in CR2), who underwent allo-HSCT, were analyzed. Twenty patients (10 in CR1, 9 in CR2, 1 in CR3) were transplanted from a matched sibling donor (MSD) and 11 patients (7 in CR1 and 4 in CR2) from a matched unrelated donor (MUD). Prior to the allo-HSCT, all the children were conditioned with FTBI at a total dose of 12 Gy with the dose reduction in the lungs to 9 Gy and with VP-16 at a single dose of 60 mg/kg intravenously. In addition, before MUD-HSCT an anti-thymocyte globulin was given for in vivo T cell depletion. Children’s demographics and transplant procedure characteristics are presented in Table 1. For graft-versus-host-disease (GvHD) prophylaxis, cyclosporine (CsA) was administered alone in patients transplanted from a MSD or in combination with “short” methotrexate (10 mg/m^2^ intravenously on days +1, +3, and +6 followed by calcium folinate 15 mg/m^2^ intravenously 24 h after each dose of methotrexate) in patients transplanted from a MUD. CsA administration started 36 h before the transplantation (day −1) and was continued intravenously until the patient could have tolerated the oral form of the drug. CsA dosage was adjusted to maintain a whole blood level of 100 ± 10 ng/mL after MSD-HSCT and 150 ± 10 ng/mL after MUD-HSCT (Chrzanowska et al. 2011).

**Table 1:** Patient’s and HSCT characteristics

Sex	
Female	*n* = 12 (39 %)
Male	*n* = 19 (61 %)
Age (years)	
Mean ± SD	11 ± 4
Range	6–18
Body weight (kg)	
Mean ± SD	43 ± 18
Range	17–80
Body surface (m^2^)	
Mean ± SD	1.28 ± 0.34
Range	0.70–2.00
Allo-HSCT indication	
ALL	29 (94 %)
NHL	2 (6 %)
Complete remission	
1	17 (55 %)
2	13 (42 %)
3	1 (3 %)
Donor type	
MSD	20 (65 %)
MUD	11 (35 %)
Stem cell source	
Bone marrow	17 (55 %)
Peripheral blood	14 (45 %)
Day of VP-16 administration	
−3	11 (35 %)
−4	20 (65 %)

*ALL* acute lymphoblastic leukemia, *allo*-*HSCT* allogeneic stem cell transplantation, *VP*-*16* etoposide, *MSD* matched sibling donor, *MUD* matched unrelated donor, *NHL* non-Hodgkin’s lymphoma

In all children included in the study, the biochemical parameters determined on the day of VP-16 administration and on the day of allo-HSCT and 3 days after the HSCT were within the respective reference norms.

#### Treatment Outcome Measures

The outcome measures were as follows: (1) engraftment (absolute neutrophil count ≥0.5 × 10^9^/L for three consecutive days and/or platelet count ≥20 × 10^9^/L for three consecutive days without any transfusion of blood platelets during the previous 7 days), (2) graft failure, (3) relapse incidence, (4) leukemia-free survival (Wachowiak 2012).

#### Analytical Procedure

Blood samples were collected into heparinized tubes before VP-16 infusion (blank plasma), at the end of the infusion and subsequently at 2, 4, 8, 24, 48, 60, 72, 96, and 120 h after the end of the infusion. The samples were centrifuged to obtain plasma, immediately frozen and kept at −20 °C until analysis. VP-16 plasma concentrations were determined using the HPLC–UV method as previously described (Chrzanowska et al. 2011). The lowest limit of detection and the lowest limit of quantitation were 0.05 and 0.1 μg/mL, respectively.

#### Pharmacokinetic and Statistical Calculations

VP-16 concentration on the allo-HSCT day was the concentration determined 72 and 96 h after the end of the infusion in 11 and 20 children, respectively. VP-16 pharmacokinetic parameters were calculated based on a three-compartment model using TopFit 2.0 software. The following VP-16 pharmacokinetic parameters were calculated: mean residence time (MRT_tot_), the central compartment volume (*V*_c_), area under the time-concentration curve (AUC), maximal concentration (*C*_max_), CL_s_, half-lives (*t*_1/2_) of α, β and γ phases, the rate constants including drug distribution from the central compartment to the second compartment (*k*_12_), drug redistribution from the second compartment to the central compartment (*k*_21_), drug distribution from the central compartment to the third compartment (*k*_13_), drug redistribution from the third compartment to the central compartment (*k*_31_), drug elimination from the central compartment (*k*_10_). The CL_s_ (mL/min) values were normalized to the children weight (mL/min/kg) as well as to the children body surface area (mL/min/m^2^). The values of *V*_c_ (L) were normalized to the body weight (L/kg). The maximum total VP-16 dose was 3600 mg (1800 mg/m^2^); therefore, for the three children with the largest body weight, who were given lower doses, the pharmacokinetic parameters were normalized to a dose of 60 mg/kg. Interpatient variability was expressed as a coefficient of variation (CV %).

Statistical analysis was performed using Statistica software version 10.0 (StatSoft, Cracow, Poland). The *p* < 0.05 was considered significant. The data are expressed as mean ± SD. Normality was determined by the Shapiro–Wilk test. The differences between groups were tested using the Student’s *t* test and the Mann–Whitney test for normally and non-normally distributed data, respectively. The correlations of the data were tested using Pearson or Spearman correlation analyses for normally and non-normally distributed data, respectively. The pLFS was calculated for the children with engraftment (*n* = 29) using the Kaplan–Meier method. Log-rank test was used to compare the distribution of pLFS within two groups (according to CR and VP-16 presence in plasma on allo-HSCT day).

### In Vitro Studies

#### VP-16 Influence on the Proliferation Rate of the Peripheral Blood Lymphocytes

In the first set of the experiments, VP-16 influence on the proliferation activity of normal lymphocytes was tested. For this purpose, lymphocytes, freshly isolated from blood by Ficoll-Paque centrifugation, were re-suspended in RPMI supplemented with 10 % fetal bovine serum (FBS), stimulated with soluble antibodies: anti-CD3 and anti-CD28 (1.0 μg/mL) and then incubated with different VP-16 concentrations. Two experiments with two different times of VP-16 exposition (24 and 72 h) were conducted. In the first experiment, VP-16 was removed after 24 h and subsequently 3-day stimulation of the lymphocytes with the antibodies without VP-16 was performed. In the other experiment, VP-16 was present in the culture medium for all the incubation time (3 days of the antibodies stimulation). The lymphocytes were harvested and beta-radiation after [3H]-thymidine incorporation was counted as a measure of cell proliferation using Wallac Micro Beta scintillation counter (Perkin Elmer).

#### The Influence of Short-Time VP-16 Exposition on the Proliferation Activity of the Peripheral Blood Lymphocytes

In order to test if the VP-16 exposition time shorter than 24 h influences the proliferation activity of lymphocytes after anti-CD3/CD28 stimulation, the lymphocytes were exposed to VP-16 for 8, 4 and 2 h. Different VP-16 concentrations (0.1, 0.3, 0.6 and 1.0 μg/mL) were used. After incubation with VP-16, the cells were washed with PBS and the three-day proliferation assay was set up. During the test, the growth of the cells was stimulated with anti-CD3/CD28 antibodies (1.0 μg/mL). [3H]-thymidine (1 mCi per well) was added to each well 6 h before the end of the test. The lymphocytes were harvested and beta-radiation was counted as a measure of cell proliferation using Wallac Micro Beta scintillation counter (Perkin Elmer).

#### VP-16 Exposure Influence on the Th1/Th2 Cytokine Responses (Cytokine Release Assay)

Cytokine bead assay (CBA approach) was used in order to test if VP-16 influences the cytokine production (Th1 versus Th2) by stimulated lymphocytes on consecutive days after VP-16 exposure. Isolated lymphocytes were cultured in 0.2 mL RPMI with 10 % FBS in 96-well plates with or without VP-16. Different concentrations of VP-16 (0.03, 0.1 and 6.25 μg/mL) were tested. After 48, 72, 96 h and seven days of the stimulation, the supernatants were harvested and tested for interleukin (IL)-6, IL-10, tumor necrosis factor (TNF)-α and interferon (IFN)-γ by Human Th1/Th2 Cytokine Cytometric Bead Array (BD Biosciences, San Jose, CA, USA). The detection was performed with flow cytometer FACSCanto (BD Biosciences, San Jose, CA, USA).

#### VP-16 Influence on the Cytotoxic Activity of the Peripheral Blood Lymphocytes

To test the influence of VP-16 on cytotoxic activity of lymphocytes we used JAM assay. Effector lymphocytes were firstly stimulated and incubated with VP-16 either for 24 or 72 h and mixed with the target tumor cell line in different ratios. In the first experiment, the effector cells were stimulated in vitro with the soluble anti-CD3/CD28 antibodies and cultured with 0.1 μg/mL VP-16 for 3 days. In the other experiment, VP-16 was washed out after 24 h exposition followed by 48 h stimulation with the antibodies. The target cells were P815 mastocytoma cell line which functions in a cell-mediated cytotoxicity manner. One day before the test, the target cells were incubated with [3H]-thymidine (10 mCi per 1 mL) overnight, then coated with the soluble anti-CD3 antibody for 1 h and washed. The assay was performed on V-shaped 96-well plate. The target cells were co-cultured with the stimulated lymphocytes at a particular effector/target ratio (25:1, 12.5:1, 6.25:1, 3:1, 1.5:1, 0.75:1) for 6 h. The lymphocytes were subsequently harvested (using Perkin Elmer Cell Harvester) and the radioactivity was measured using a Wallac Micro Beta scintillation counter (Perkin Elmer).

## Results

### Clinical Studies

#### The VP-16 Concentrations on the Allo-HSCT Day

In 26 children (84 %), VP-16 was detectable in the plasma samples at concentrations ranging 0.1–1.5 μg/mL (mean ± SD: 0.4 ± 0.4 μg/mL) 72 h after the end of VP-16 infusion. In the plasma samples collected 96 h after the end of the infusion, VP-16 was detectable in 12 children (39 %) at concentrations 0.1–0.9 μg/mL (mean ± SD: 0.4 ± 0.2 μg/mL) (Table 2).

**Table 2:** VP-16 concentrations 48, 72, 96 and 120 h after the end of infusion in all children and according to the VP-16 administration day

*n* = 31	VP-16		Lack of blood sample	VP-16 concentration (μg/mL)	
	Detectable	<LOQ		Mean ± SD	Range
48 h	30 (97 %)	0 (0 %)	1 (3 %)	0.8 ± 0.6	0.1–2.4
72 h	26 (84 %)	5 (16 %)	0 (0 %)	0.4 ± 0.4	0.1–1.5
96 h	12 (39 %)	18 (58 %)	1 (3 %)	0.4 ± 0.2	0.1–0.9
120 h	7 (23 %)	12 (39 %)	12 (39 %)	0.3 ± 0.1	0.1–0.5
VP-16 administration day −3 (*n* = 11)					
48 h	11 (100 %)	0 (0 %)	0 (0 %)	0.8 ± 0.4	0.2–1.5
72 h (allo-HSCT day)	10 (91 %)	1 (9 %)	0 (0 %)	0.4 ± 0.3	0.1–0.8
96 h	3 (27 %)	7 (64 %)	1 (9 %)	0.5 ± 0.1	0.3–0.6
120 h	2 (18 %)	5 (45 %)	4 (36 %)	0.2 ± 0.1	0.1–0.3
VP-16 administration day −4 (*n* = 20)					
48 h	19 (95 %)	0 (0 %)	1 (5 %)	0.8 ± 0.7	0.1–2.4
72 h	16 (80 %)	4 (20 %)	0 (0 %)	0.5 ± 0.4	0.1–1.5
96 h (allo-HSCT day)	9 (45 %)	11 (55 %)	0 (0 %)	0.3 ± 0.3	0.1–0.9
120 h	5 (25 %)	7 (35 %)	8 (40 %)	0.3 ± 0.1	0.2–0.5

*allo*-*HSCT* allogeneic stem cell transplantation, *LOQ* limit of quantification, *VP*-*16* etoposide

On the allo-HSCT day, VP-16 was detectable in the plasma samples of 19 children (mean ± SD: 0.4 ± 0.3 μg/mL). VP-16 at concentrations of 0.1–0.8 μg/mL (mean ± SD: 0.4 ± 0.3 μg/mL) was detectable in 10 children who had been subjected to allo-HSCT 3 days after the VP-16 administration, and at concentrations of 0.1–0.9 μg/mL (mean ± SD: 0.3 ± 0.3 μg/mL) in 9 children for which allo-HSCT was performed 4 days after the VP-16 administration (Table 2).

#### The Correlations between the VP-16 Concentrations and the Pharmacokinetic Parameters

**Fig. 1:**
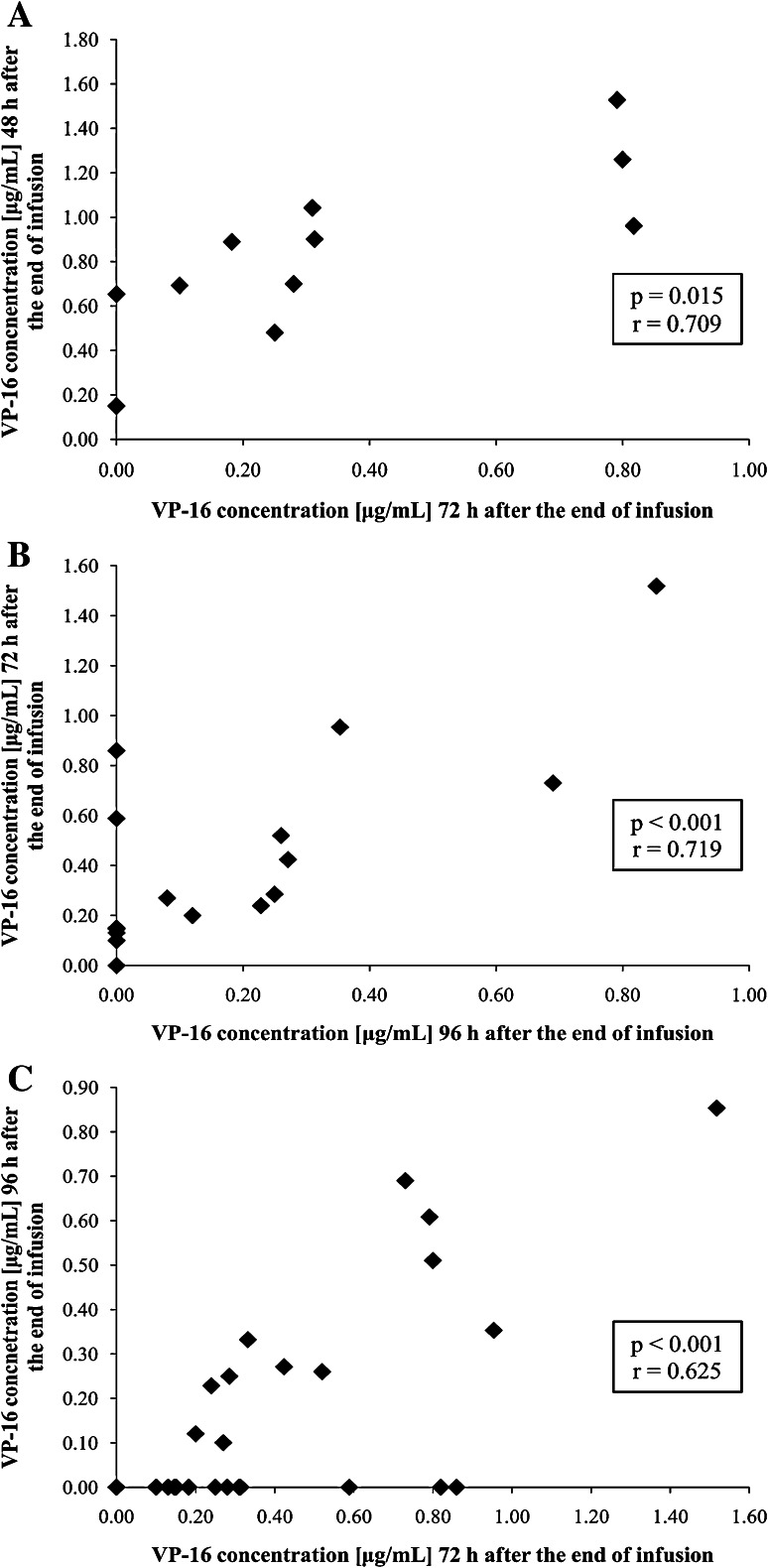
The correlations between the etoposide (VP-16) concentrations determined 24 h before allo-HSCT and on the allo-HSCT day [48 and 72 h after the end of the infusion for the children with VP-16 administered on day −3 (**a**), 72 h and 96 h after the end of the infusion for the children with VP-16 administered on day −4 (**b**)] and between the VP-16 concentrations determined 72 h after the end of the infusion and 96 h after the end of the infusion for all children included in the study (**c**)

To verify whether the determination of VP-16 concentration 24 h before the planned allo-HSCT may predict the concentration of VP-16 on the allo-HSCT day, we checked the correlations between the VP-16 concentrations determined 24 h prior to allo-HSCT and the VP-16 concentrations on the allo-HSCT day. In children with VP-16 administered on day –3, the drug concentration determined 48 h after the end of the infusion correlated significantly with the VP-16 concentrations on the allo-HSCT day, i.e. 72 h after the end of the infusion (*p* = 0.015; Fig. 1a). In children with VP-16 administered on day –4, the drug concentration determined 72 h after the end of the infusion also correlated significantly with the VP-16 concentration on the allo-HSCT day, i.e. 96 h after the end of the infusion (*p* < 0.001; Fig. 1b). For all children included in the study, VP-16 concentrations determined 72 h after the end of the infusion showed a significant correlation with the concentrations determined 96 h after the end of the infusion (*p* < 0.001; Fig. 1c).

The concentration of VP-16 determined 72 h after the end of the infusion correlated positively with the MRT_tot_, AUC and *t*_1/2_ γ and negatively with the CL_s_ (mL/min/kg) and *k*_31_ constant, whereas the correlation with the CL_s_ normalized to the body surface was close to significant (*p* = 0.055). The concentration of VP-16 determined 96 h after the end of the infusion correlated positively with the MRT_tot_, *t*_1/2_ α and γ and negatively with the *k*_21_ and *k*_31_ constants. The concentration of VP-16 on the allo-HSCT day correlated positively with the MRT_tot_, AUC and *t*_1/2_ γ and negatively with the CL_s_ (mL/min/kg) and *k*_31_ constant (Table 3).

**Table 3:** The correlations between VP-16 pharmacokinetic parameters and VP-16 concentrations determined 72, 96 h after the end of infusion and on allo-HSCT day

Parameter	VP-16 concentration (μg/mL)					
	72 h		96 h		Allo-HSCT day	
	*r*	*p*	*r*	*p*	*r*	*p*
MRT_tot_ (h)	0.780	<0.001	0.666	<0.001	0.614	<0.001
*V*_c_ (L/kg)	−0.136	0.466	−0.262	0.154	−0.209	0.259
CL_s_ (mL/min/kg)	−0.414	0.021	−0.241	0.192	−0.365	0.043
CL_s_ (mL/min/m^2^)	−0.349	0.055	−0.278	0.129	−0.273	0.137
AUC (µg/mL·h)	0.420	0.019	0.252	0.171	0.362	0.045
*C*_max_ (µg/mL)	0.120	0.519	0.234	0.205	0.182	0.328
*t*_1/2_ α (h)	0.207	0.265	0.399	0.026	0.223	0.228
*t*_1/2_ β (h)	0.303	0.098	0.179	0.335	0.181	0.330
*t*_1/2_ γ (h)	0.628	<0.001	0.720	<0.001	0.597	<0.001
*k*_12_ (1/h)	0.051	0.786	−0.170	0.362	−0.054	0.771
*k*_21_ (1/h)	−0.208	0.262	−0.439	0.014	−0.210	0.256
*k*_13_ (1/h)	0.263	0.153	0.174	0.348	0.216	0.243
*k*_31_ (1/h)	−0.614	<0.001	−0.709	<0.001	−0.583	0.001
*k*_10_ (1/h)	−0.283	0.123	−0.079	0.672	−0.175	0.345

#### The Correlations between the VP-16 Concentrations and the Children’s Characteristics

VP-16 concentrations determined at 72, 96 h and on the allo-HSCT day did not correlate with the children’s characteristics such as their age, body surface and body weight as well as total VP-16 dose (data not shown).

The mean VP-16 concentration on the allo-HSCT day was higher in the boys (0.3 ± 0.3 μg/mL) than in the girls (0.1 ± 0.2 μg/mL) and the difference was statistically significant (*p* = 0.047). At the same time, the girls and the boys included in the study did not differ in total VP-16 dose, age, the body weight and the body surface area.

Additionally, the VP-16 concentrations (determined 72, 96 h and on the allo-HSCT day) did not differ regarding the type of hematopoietic stem cell donor (MSD or MUD) as well as the prophylactic administration of the antifungal drugs (itraconazole, fluconazole, posaconazole, voriconazole).

Out of 12 children with VP-16 not detectable on HSCT day, the acute GvHD grade II–IV was observed in eight (67 %) (II^0^, *n* = 3; III^0^, *n* = 4; IV^0^, *n* = 1), while among 19 children with detectable VP-16 on HSCT day the acute GvHD grade II–IV occurred in nine (47 %) (II^0^, *n* = 6; III^0^, *n* = 3; *p* = 0.307).

#### The Correlations between the VP-16 Concentrations and the Time of Engraftment

In all children included in the study, the time of engraftment did not differ between children with detectable and undetectable VP-16 on the allo-HSCT day. In the children with VP-16 undetectable on the allo-HSCT day, the granulocytes and platelets recovery were observed on days 17 ± 7 and 25 ± 28, respectively. In the children with VP-16 detectable on the allo-HSCT day, the granulocytes and platelets recovery were observed on days 16 ± 5 and 24 ± 14, respectively. There were also no differences in the time of engraftment regarding VP-16 concentrations on the allo-HSCT day (detectable or undetectable), CR (CR1 or CR2) or the source of stem cells (bone marrow or peripheral blood). Moreover, none of the VP-16 concentrations (72, 96 h, on the allo-HSCT day) correlated with the day of granulocytes and platelets recovery (data not shown).

#### The ALL Relapse and pLFS

ALL relapse occurred within 4–17 months after the allo-HSCT (median 10 months) in 4 out of 29 children. Two children were in CR1 and two in CR2 before the allo-HSCT. Three per 20 (15 %) children were transplanted from MSD and 1/11 (9 %) from MUD. Two per 17 (12 %) children received the stem cells from bone marrow and 2/14 (14 %) from peripheral blood.

In all 4 children with ALL relapse, VP-16 was detectable in plasma on the allo-HSCT day in concentrations ranging 0.1–0.8 μg/mL (mean ± SD: 0.3 ± 0.3 μg/mL). In these children, the VP-16 concentrations 24 h before the allo-HSCT ranged 0.1–1.5 μg/mL (mean ± SD: 0.7 ± 0.6 μg/mL). In the children with VP-16 undetectable on the allo-HSCT day, no ALL relapse occurred, although the values of the mean VP-16 concentrations (72, 96 h after the end of the infusion and on the allo-HSCT day) did not differ between the children with and without ALL relapse.

**Fig. 2:**
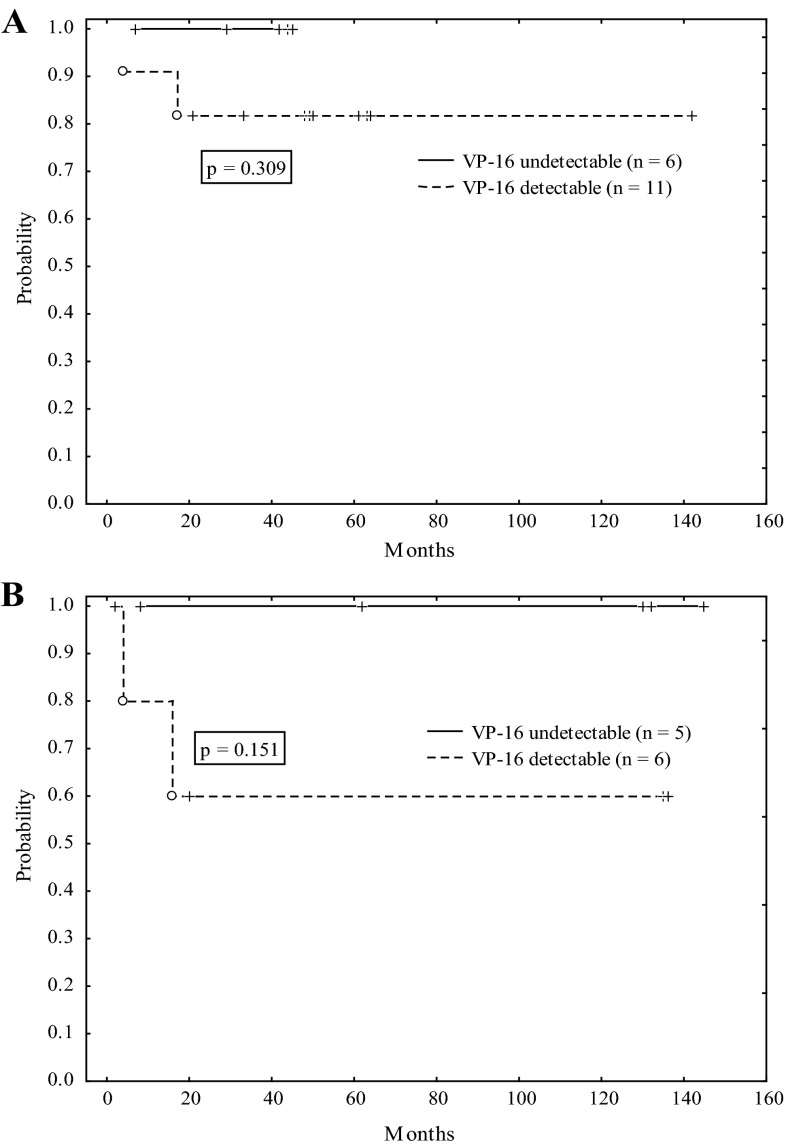
The comparison of probability of leukemia-free survival (pLFS) after allo-HSCT between the children with detectable and undetectable etoposide (VP-16) on the allo-HSCT day for the children in the first complete remission (CR1; **a**) and the second complete remission (CR2; **b**)

For the children who were in CR1, 4-year pLFS was 100 and 82 % for VP-16 undetectable (*n* = 6) and detectable (*n* = 11) in plasma on the allo-HSCT day, respectively (Fig. 2a); however, the difference was not statistically significant (*p* = 0.309). For the children who were in CR2, 11-year pLFS was 100 and 60 % for VP-16 undetectable (*n* = 5) and detectable (*n* = 6) in plasma on the allo-HSCT day, respectively (Fig. 2b). Also, in this case the difference was insignificant (*p* = 0.151). The difference in the follow-up time for the children in CR1 and CR2 was due to a later inclusion of the children in CR1 to the allo-HSCT.

### In Vitro Studies

#### VP-16 Inhibited the Proliferation Rate of the Peripheral Blood Lymphocytes, Which was Correlated With the Time of Exposition

**Fig. 3:**
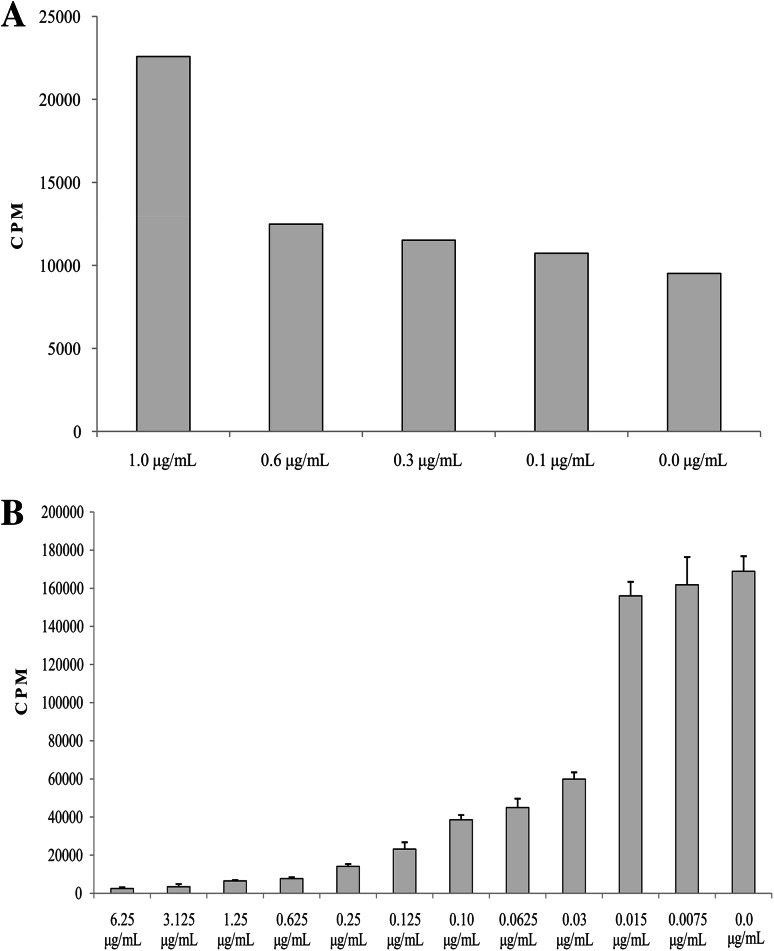
Proliferation rate of the peripheral blood lymphocytes after 24 h (**a**) and 72 h (**b**) incubation with etoposide (VP-16) in various concentrations. *CPM* counts per minute

The results of the first set of experiments showed that both 24 h (Fig. 3a) and 72 h (Fig. 3b) incubation with VP-16 influenced the proliferation activity of the stimulated lymphocytes in a concentration-dependent manner. We observed that the cell proliferation inhibition started at VP-16 concentration of 0.03 μg/mL and reached the maximum at 0.6 μg/mL.

#### Short-Time VP-16 Exposition did not Affect the Proliferation Activity of the Peripheral Blood Lymphocytes

**Fig. 4:**
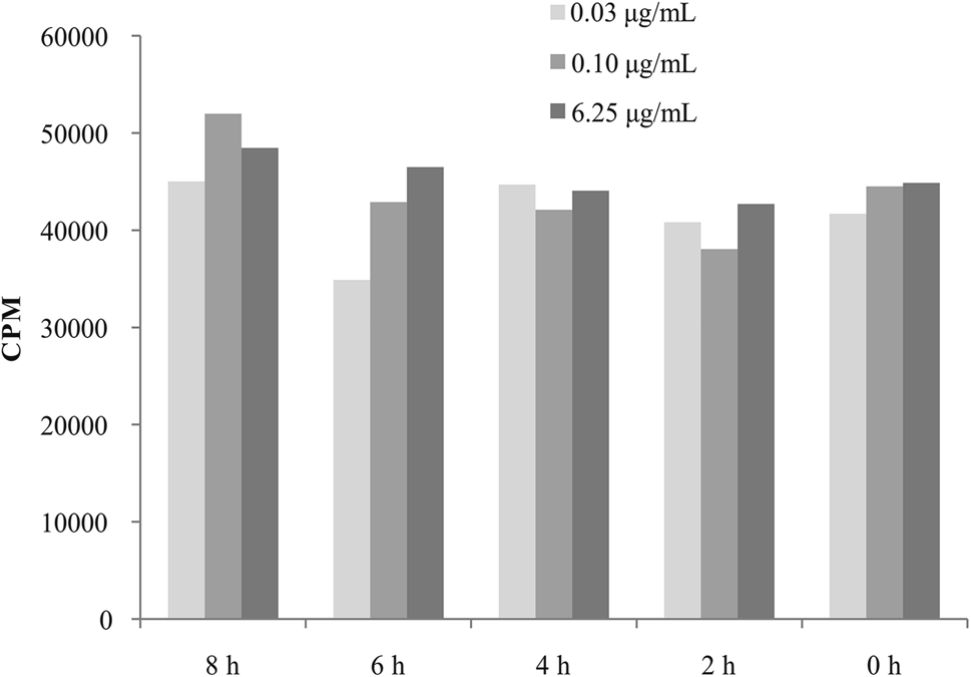
The influence of short-time exposition of various etoposide (VP-16) concentrations on the proliferation activity of the peripheral blood lymphocytes. *CPM* counts per minute

We demonstrated that the incubation with different VP-16 concentrations for a time shorter than 24 h did not affect the proliferation of lymphocytes (Fig. 4).

#### VP-16 Exposure did not Affect the Th1/Th2 Cytokine Responses (Cytokine Release Assay)

**Fig. 5:**
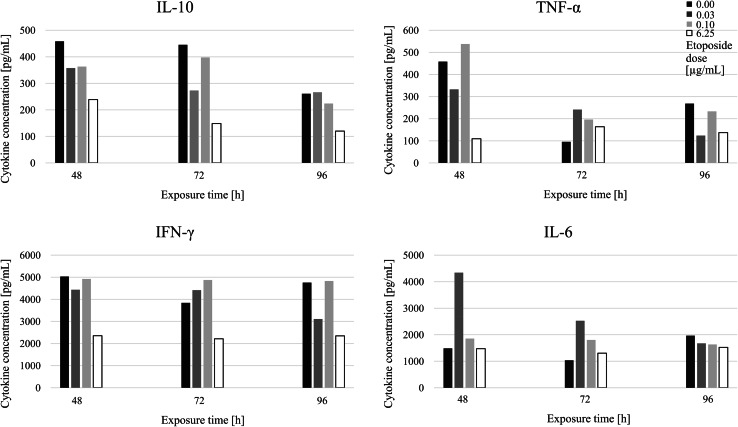
The influence of different etoposide (VP-16) concentrations (0.0, 0.03, 0.1 and 6.25 μg/mL) and the exposure time (48, 72, 96 h) and on the cytokine (IL-6, IL-10, TNF-α and IFN-γ) production

In the CBA approach, the VP-16 influence on the cytokine production (Th1 versus Th2) was tested. As it is shown in Fig. 5, we observed no significant differences in the effect of VP-16 exposition on the cytokine production by the stimulated lymphocytes. After 7 days of the stimulation, IL-6, IL-10, TNF-α and IFN-γ concentrations were decreasing (data not shown); however, this trend was not significant. Our results showed that the highest VP-16 concentration (6.25 μg/mL) decreased the IFN-γ (Th1 cytokine) and IL-10 (Th2 cytokine) production comparing to the non-treated lymphocytes and the effect was consistent in the first days of the experiment. However, this VP-16 concentration was shown to be the highest concentration which let the cells to survive, thus the influence on the cytokine production was more likely due to significant inhibition of their proliferation rate. These results indicate that VP-16 did not considerably modulate Th1/Th2 responses in the lymphocytes.

#### VP-16 did not Influence the Cytotoxic Activity of the Peripheral Blood Lymphocytes

**Fig. 6:**
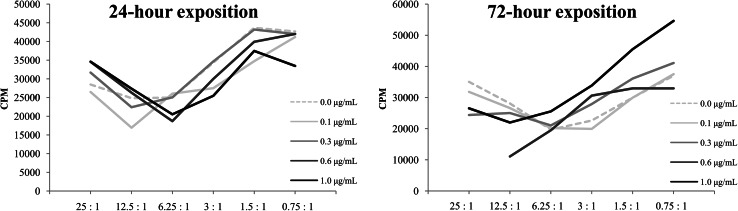
Cytotoxic activity of the peripheral blood lymphocytes after 24 and 72 h exposition to various concentrations of etoposide (VP-16). Target cells were co-cultured with stimulated lymphocytes at a particular effector/target ratio: 25:1; 12.5:1; 6.25:1; 3:1; 1.5:1; 0.75:1 for 6 h. *CPM* counts per minute

Our data showed that VP-16 did not affect the cytotoxicity of the lymphocytes since no significant differences in the cytotoxic activity were observed in the VP-16-treated and non-treated lymphocytes. Those results were observed either in the continuous presence of VP-16 during the lymphocytes stimulation or after the 24 h exposition to VP-16 (Fig. 6).

## Discussion

Optimization of the conditioning regimen in children undergoing allo-HSCT for ALL is still needed and, among others, the impact of the VP-16 presence in plasma on the allo-HSCT day on the transplantation long-term results as well as the safe level of VP-16 concentration, which would make no toxic damage to the allogeneic hematopoietic cells and to the immune cells, responsible for GvL effect, present in the transplanted material are not determined. According to the literature data, there were some attempts, but they have not reached conclusive findings (Krüger et al. 1995). According to some authors, HSCT should not be performed before a total elimination of the cytotoxic drugs used in conditioning, so that the transplanted hematopoietic stem cells are not affected by these agents (Mross et al. 1994). According to the literature, high plasma VP-16 concentrations persisting for a long time may negatively affect the engraftment (Hartmann and Lipp 2006). Therefore, in this study, we comprehensively evaluated the importance of VP-16 concentrations determined 72 and 96 h after the end of VP-16 infusion.

According to Blume et al. (1987), other authors (Dopfer et al. 1991; Jamieson et al. 2003; Mehta and Davies 2008; Schrauder et al. 2008) and the ALL-SCT I-BFM protocol (Peters et al. 2015) used in studied patients, allo-HSCT should be performed 72 h after the end of VP-16 infusion. However, at that time, VP-16 was present in plasma in 84 % of the children at concentrations 0.1–1.5 μg/mL. In majority of the studied children (65 %), allo-HSCT was performed 4 days after the drug infusion as a consequence of our own preliminary VP-16 pharmacokinetic results, and on this day VP-16 was still detectable in 39 % of children at concentrations between 0.1 and 0.9 μg/mL. Detectable VP-16 was also observed in 37 % of the children’s plasma samples collected 120 h after the end of its infusion, i.e. on day +1 or day +2 after allo-HSCT.

In order to optimize the VP-16 dosing and determine the factors that could affect the results of allo-HSCT, we correlated the concentrations observed 72 and 96 h after the end of VP-16 infusion and on the allo-HSCT day with VP-16 pharmacokinetic parameters and the children’s demographic data. VP-16 *C*_max_ observed at the end of the infusion did not correlate with the concentrations determined 72 and 96 h after the infusion and on the allo-HSCT day. Therefore, *C*_max_ appears not to be a useful parameter in predicting the prolonged VP-16 elimination. The positive correlations between the VP-16 concentrations determined 72 h after the end of the infusion and the AUC, and the negative one with the CL_s_ (mL/min/kg) and close to significant with the CL_s_ (mL/min/m^2^) indicate that the concentration of VP-16 observed 72 h after the infusion in children participating in the study may be high enough to adversely affect the engraftment.

The positive correlations between VP-16 concentrations (determined 72 and 96 h after the end of the infusion and on the allo-HSCT day) and the MRT_tot_ and *t*_1/2_ γ as well as the negative correlations with the *k*_31_ constant explained the long-lasting VP-16 concentrations in children’s plasma, despite high values of VP-16 CL_s_.

The significant positive correlations observed between the VP-16 concentrations determined 24 h prior to allo-HSCT and on the allo-HSCT day may be helpful in predicting the concentration of VP-16 in plasma on the allo-HSCT day. In the case of VP-16 administration on day −4, the drug concentration determined one day before the allo-HSCT (day −3) may indicate that on the allo-HSCT day VP-16 may still be detectable in plasma due to its prolonged elimination.

Based on our results, we assumed that VP-16 presence in plasma on the allo-HSCT day may impair the function of the donor lymphocytes present in the transplant material, which are responsible for the GvL effect, as well as affect the long-term results of the transplantation as ALL relapse occurred in the children with VP-16 detectable in plasma on the allo-HSCT day. At the same time, there were no significant differences in the values of VP-16 pharmacokinetic parameters between the children with and without ALL relapse. However, a tendency toward a higher CL_s_ (mL/min/m^2^) in the children with relapsed ALL was observed. It confirmed the observation that VP-16 should not be eliminated too quickly as its long-lasting low concentrations may be responsible for the treatment response (Rodman et al. 1994). Krüger et al. (1995) in vitro studies showed that VP-16 concentration which is safe to perform HSCT should be lower than 0.40 μg/mL (Rodman et al. 1994; Littlewood et al. 1985). In our study, among 19 children who had VP-16 detectable in plasma on the allo-HSCT day, VP-16 concentrations were greater than 0.4 μg/mL in 6 children (32 %). ALL relapse, however, was also observed in the children with VP-16 concentration lower than 0.4 μg/mL. It must be stated that the observation was made on the small group of patients and those with relapses formed also too small group to draw firm conclusions.

We also assessed in vitro whether the VP-16 at concentration, observed on the allo-HSCT day, may influence the engraftment as, according to our knowledge, the influence of VP-16 concentrations observed on allo-HSCT day on the transplanted hematopoietic stem cells as well as on the GvL effector cells has not been studied yet. The results showed that VP-16 influenced the proliferation activity of the stimulated lymphocytes depending on the VP-16 concentration and the incubation time.

Based on the results, it may be assumed that in the presence of VP-16 for 24 h, the intracellular defense mechanisms were triggered, resulting in the increased lymphocytes proliferation in the samples with VP-16 concentration of 1 μg/mL than in the control samples. The 72 h exposure was likely to be long enough to exhaust the potential of defense mechanisms what allowed VP-16 to reveal its cytotoxic effect. It is proved that the treatment programs including prolonged administration of VP-16 had better efficiency due to the long-lasting DNA breaks whereas VP-16 exposure shorter than 24 h resulted in a reduced response to the treatment (Hande 1998) what is in accordance with our results.

Based on the results obtained after 72 h exposure, we concluded that the minimum VP-16 concentration needed to arrest the growth of the lymphocytes, stimulated with anti-CD3 and anti-CD28, was 0.03 μg/mL. The results of in vitro studies cannot be extrapolated directly to in vivo; however, they suggest that the VP-16 presence in plasma samples of children conditioned to allo-HSCT, at concentrations showed in our study, may influence to some extent the transplanted hematopoietic stem cells as well as the effector cells of the GvL effect. The results of the clinical trials are in accordance with our observation as the VP-16 concentrations of 0.5–2.0 μg/mL are considered anti-cancer active (Hande 1998). On the other hand, in vitro studies of Tazawa et al. (2012) showed no effect of VP-16 low concentrations (0.5 and 1 μg/mL) on the ability of the leukemic cell line K-562 to survive; however, the study included myeloid cell line.

In summary, our results demonstrate the interpatient variability and the presence of detectable VP-16 plasma concentrations 72 h after its infusion in significant proportion of children with ALL conditioned for allo-HSCT with FTBI and high-dose VP-16. In addition, the results of the study suggest that VP-16 presence in plasma on the day of allo-HSCT may be considered as one of the factors increasing the risk of post-transplant ALL relapse, which remains the most frequent cause of the treatment failure in children transplanted for ALL. Thus, there is a need to monitor VP-16 levels and if 72 h after the end of VP-16 administration the plasma concentration is still above 0.1 μg/mL, then the postponement of transplantation for the next 24 h should be considered to protect GvL effector cells from transplant material.
